# PARACENTRAL ACUTE MIDDLE MACULOPATHY ASSOCIATED WITH BRANCH RETINAL ARTERY OCCLUSION DUE TO POLYCYTHEMIA IN A PATIENT WITH TETRALOGY OF FALLOT

**DOI:** 10.1097/ICB.0000000000001054

**Published:** 2020-09-18

**Authors:** Nurullah Koçak, Bilgehan Erduran, Mustafa Subaşı, Volkan Yeter

**Affiliations:** Department of Ophthalmology, Ondokuzmayıs University Hospital, Atakum, Turkey.

**Keywords:** branch retinal artery occlusion, paracentral acute middle maculopathy, polycythemia, tetralogy of Fallot

## Abstract

To describe the occurrence of paracentral acute middle maculopathy (PAMM) associated with branch retinal artery occlusion secondary to polycythemia in a patient with tetralogy of Fallot.

**Methods:**

Case report.

**Results:**

A 30-year-old man presented with acute vision loss and superior visual deficit in his left eye for two days. His medical record had a tetralogy of Fallot. Complete blood count showed an erythrocyte count of 9.88 million/µL (4.4–5.6), hemoglobin of 17.7 g/dL (13.5–16.9), and hematocrit of 65.4% (40–49). The best-corrected visual acuity was 20/25 in the left eye, and a diagnosis of left inferotemporal branch retinal artery occlusion was made. Spectral-domain optical coherence tomography revealed a characteristic hyperreflective band-like lesion on the inner nuclear layer consistent with PAMM.

**Conlusion:**

Polycythemia may be a trigger for branch retinal artery occlusion–associated PAMM. We suggest a new precursor cause of PAMM that is previously undescribed.

## Abstract 2

We aim to present a case with paracentral acute middle maculopathy associated with branch retinal artery occlusion secondary to polycythemia, which is suggested a new precursor cause of paracentral acute middle maculopathy. We believe this report will be of interest to the readers of your journal.

Paracentral acute middle maculopathy (PAMM) is a recently recognized entity that was first described by Sarraf et al in 2013.^1^ Paracentral acute middle maculopathy is characterized by a hyperreflective band-like lesion around the inner nuclear layer (INL) on optical coherence tomography (OCT) that is caused by ischemia of intermediate and/or deep retinal capillary plexus.^2^ Patients present with acute nonspecific vision loss, paracentral scotoma, and altitudinal visual field defects.

Paracentral acute middle maculopathy has been associated with different retinal vascular diseases and systemic diseases/factors that include central retinal vein occlusion, diabetic retinopathy, branch or central retinal artery occlusion, sickle cell retinopathy, Purtscher retinopathy, migraine, primary antiphospholipid syndrome, oral contraceptive pills, and medical procedures.^3–8^ In one series, the most common referring diagnoses were branch retinal artery occlusion (BRAO) and central retinal vein occlusion with or without cilioretinal artery occlusion.^9^ In this report, we describe a case of PAMM secondary to BRAO in a patient with a history of tetralogy of Fallot (TOF). Tetralogy of Fallot is the most common congenital cyanotic heart disease in children caused by a combination of four heart defects: pulmonary valve stenosis, ventricular septal defect, overriding aorta, and right ventricular hypertrophy and causes hypoxia-related secondary polycythemia. Cyanotic congenital heart diseases with secondary polycythemia and hyperviscosity state are associated with a reduction in blood flow, stagnation of blood, and thrombosis. To the best of our knowledge, PAMM has never been reported with secondary polycythemia.

## Case Report

**Fig. 1.**
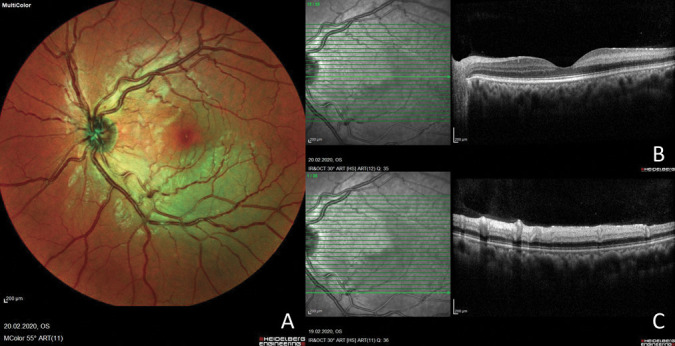
**A.** Multicolor fundus photograph of the left eye showing a deep retinal whitening area around the inferotemporal arcade. **B.** Optical coherence tomography (a horizontal slab on the fovea) of the left eye showing a hyperreflective band on the level of the INL consistent with PAMM. **C.** Spectral-domain OCT through the central area of the infarct displays ischemia and hyperreflectivity of both the middle and inner retinal layers.

**Fig. 2.**
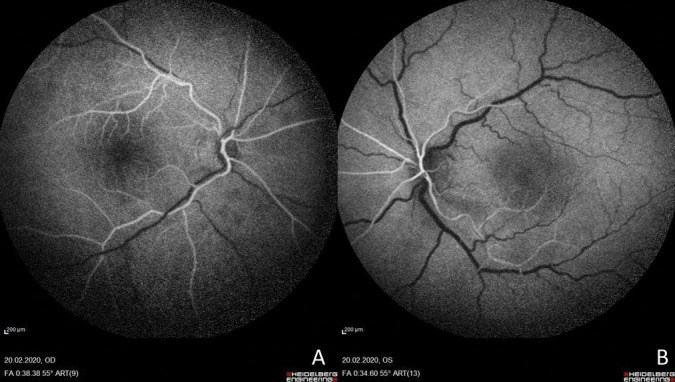
Fundus fluorescein angiography exhibits delayed filling in retinal vascular bed in both eyes (frame shown at 38 seconds).

A 30-year-old man presented with two days of painless vision loss and superior visual field deficit in his left eye. His past medical history consisted of tetralogy of Fallot, surgical correction was offered but deferred when he was a child. The patient complained of dyspnea and cyanosis with exercise and excessive physical activity. Family and social histories were noncontributory. Ophthalmological examination was performed two days after the onset of symptoms. The best-corrected visual acuity was 20/20 in his right eye and 20/25 in his left eye. Intraocular pressures were normal, and anterior segment examination was unremarkable. Clubbing and cyanosis of the fingers were noted. The fundus examination of the right eye displayed retinal venous dilatation and tortuosity in all four quadrants. A patch of deep retinal whitening was noted in the area of the inferotemporal vascular arcade of the left eye. Spectral-domain OCT (Spectralis multicolor; Heildelberg Engineering, Heildelberg, Germany) was performed and hyperreflectivity of the INL was found at the border of the infarct while both inner and middle retinal hyperreflectivity and ischemia were noted within the central region of the infarct (Figure 1). Fluorescein angiography (FA) exhibited delayed filling in retinal vascular beds in both eyes, and these findings were consistent with the slow flow in retinal circulation secondary to polycythemia. Fluorescein angiography also showed delayed filling in the infratemporal branch retinal artery (Figure 2). He had an erythrocyte count of 9.88 million/µL (4.4–5.6), hemoglobin of 17.7 g/dL (13.5–16.9), and hematocrit of 65.4% (40–49). His sodium, potassium, calcium, blood urea nitrogen, creatinine, hemoglobin electrophoresis, and rheumatologic workup were in normal limits. Hypercoagulability tests, such as platelets, factor V Leiden, protein C and S, homocysteine, anticardiolipin antibodies, and antithrombin III, were normal. A transthoracic echocardiogram was consistent with the tetralogy of Fallot, and no cardiac thrombus, mass, or vegetation was observed. Also, carotid Doppler of the neck and computed tomography of head and orbit were unremarkable. The patient consulted with hematology and cardiology department and recommended acetylsalicylic acid daily. Two months later, the final best-corrected visual acuity was 20/20 in his left eye, but the patient was still complaining of visual field deficit.

## Discussion

Paracentral acute middle maculopathy is a relatively new clinical entity that affects the intermediate and/or deep retinal capillary plexus.^1^ Paracentral acute middle maculopathy is not a specific disease but rather a clinical sign and generally associated with retinal vascular diseases, such as retinal arterial and vein occlusions, diabetic retinopathy, sickle cell retinopathy, and hypertensive retinopathy.^2,3,5^

The hypercoagulable state is a well-known risk factor for retinal arterial occlusion in young healthy adults, and etiologies include primary and secondary polycythemia, thrombocytosis, hyperhomocysteinemia, and systemic vasculitis.^10^ In the present case, the patient had a medical history of uncorrected tetralogy of Fallot and secondary polycythemia, which might have caused branch retinal arterial occlusion and PAMM by the mechanism of hyperviscosity and the slow flow in the retinal circulation. The retinal arterial occlusive disease can affect both superficial and deep capillary plexus as well as only deep capillary plexus. The presence of different levels of ischemia may be attributable to a variation in ischemic susceptibility. It is proposed that the deep capillary plexus may be more vulnerable to an ischemic insult because it may reside in a watershed region of oxygen supply. It was shown that PAMM occurs specifically at the edges rather than at the core of ischemic lesions in eyes with BRAO.^11^ We also observed PAMM lesions at the edges of the ischemic area consistent with this theory. This finding has been previously observed and may be explained by the presence of higher oxygen levels or better collateralization at the borders of the infarct adjacent to normal retinal tissue.^12^ The deep capillary plexus is predominantly a venous outflow plexus and generally affected in pathologic slow flow disorders. Besides the retinal arterial occlusion, low-flow secondary to polycythemia may contribute additionally to PAMM development. Moreover, bilaterally venous dilatation, no evidence of persistent central vein occlusion such as cotton wool spots and/or intraretinal hemorrhages, and delayed filling of vascular beds on fundus FA support our slow-flow mechanism secondary to polycythemia.

## Conclusion

Paracentral acute middle maculopathy can occur secondary to polycythemia and should be considered on the differential in any patient with visual loss and paracentral scotoma. As more cases of PAMM are identified and further characterized by improved and novel imaging methods, we expect to learn of more associated conditions. To the best of our knowledge, this is the first case presentation with PAMM secondary to polycythemia.
